# Genomic prediction based on runs of homozygosity

**DOI:** 10.1186/s12711-014-0064-6

**Published:** 2014-10-04

**Authors:** Tu Luan, Xijiang Yu, Marlies Dolezal, Alessandro Bagnato, Theo HE Meuwissen

**Affiliations:** Department of Animal and Aquacultural Sciences, Norwegian University of Life Sciences, Ås, N-1432 Norway; Dipartimento di Scienze e Tecnologie Veterinarie per la Sicurezza Alimentare, Università degli Studi di Milano, Via Celoria 10, 20133 Milano, Italy

## Abstract

**Background:**

Genomic prediction is based on the accurate estimation of the genomic relationships among and between training animals and selection candidates in order to obtain accurate estimates of the genomic estimated breeding values (GEBV). Various methods have been used to predict GEBV based on population-wide linkage disequilibrium relationships (**G**_**IBS**_) or sometimes on linkage analysis relationships (**G**_**LA**_). Here, we propose a novel method to predict GEBV based on a genomic relationship matrix using runs of homozygosity (**G**_**ROH**_). Runs of homozygosity were used to derive probabilities of multi-locus identity by descent chromosome segments. The accuracy and bias of the prediction of GEBV using **G**_**ROH**_ were compared to those using **G**_**IBS**_ and **G**_**LA**_. Comparisons were performed using simulated datasets derived from a random pedigree and a real pedigree of Italian Brown Swiss bulls. The comparison of accuracies of GEBV was also performed on data from 1086 Italian Brown Swiss dairy cattle.

**Results:**

Simulations with various thresholds of minor allele frequency for markers and quantitative trait loci showed that **G**_**ROH**_ achieved consistently more accurate GEBV (0 to 4% points higher) than **G**_**IBS**_ and **G**_**LA**_. The bias of GEBV prediction for simulated data was higher based on the real pedigree than based on a random pedigree. In the analyses with real data, **G**_**ROH**_ and **G**_**LA**_ had similar accuracies. However, **G**_**LA**_ achieved a higher accuracy when the prediction was done on the youngest animals. The **G**_**IBS**_ matrices calculated with and without standardized marker genotypes resulted in similar accuracies.

**Conclusions:**

The present study proposes **G**_**ROH**_ as a novel method to estimate genomic relationship matrices and predict GEBV based on runs of homozygosity and shows that it can result in higher or similar accuracies of GEBV prediction than **G**_**LA**_, except for the real data analysis with validation of young animals. Compared to **G**_**IBS**_, **G**_**ROH**_ resulted in more accurate GEBV predictions.

## Background

With the development of high-throughput genotyping technologies and the reduction of genotyping costs, genomic selection (GS) has become a practical and effective tool for animal and plant breeding [1,2]. In genomic selection [3], markers that densely cover the genome are expected to be in complete or partial population-wide linkage disequilibrium (LD) with the QTL (quantitative trait loci), which allows a high fraction of the genetic variance to be explained by the markers [4]. The population-wide LD information can be approximated by a relationship matrix based on identity by-state (IBS) (**G**_**IBS**_ matrix), where the relationships are reflected by the actual proportion of shared marker alleles that are IBS, as a deviation from expected IBS allele sharing in the population. With an animal model similar to the classical mixed model, best linear unbiased prediction (BLUP) of the GEBV can be achieved by replacing the pedigree-based numerator relationship matrix with the **G**_**IBS**_ matrix (G-BLUP) [5,6].

Habier et al. [7] and Luan et al. [8] found that, although genomic prediction based on IBS information does not in principle require pedigree data, it does use the family structure of the population, since the markers capture the LD that arises from the family structure. This LD allows close genetic relationships between animals within the pedigree, which are explained by linkage analysis (LA). Fernando and Grossman [9] reported a genomic identity-by-descent (IBD) matrix (**G**_**LA**_ matrix) that contains IBD probabilities within a known pedigree and that depicts this LA information. Thus, based on a limited number of generations within the known pedigree, GEBV can be predicted using the **G**_**LA**_ matrix [6].

For genomic prediction based on the **G**_**LA**_ matrix, marker alleles are IBD if they can be traced back to common ancestors in a clearly defined base generation. The probability of IBD is based only on pedigree information and the inheritance of marker alleles is traced within the pedigree. For genomic prediction based on the **G**_**IBS**_ matrix, it is not possible to identify whether IBS marker alleles are IBD or not, since there is no defined base generation, which means that the **G**_**IBS**_ matrix potentially depicts non-recorded relationships that occurred before the base generation of the known pedigree. Thus, a key difference between the **G**_**IBS**_ and **G**_**LA**_ matrices is the number of generations they take into consideration. For the **G**_**IBS**_ matrix, this is limited by the age of the SNPs used, whereas estimates of IBD coefficients with the **G**_**LA**_ matrix are based only on the known pedigree, and founders are considered to be unrelated. Because of selection, mutations that cause genetic variation in the trait of interest may be considerably younger than the mutations that underlie SNPs on the SNP chip that have a high minor allele frequency (MAF), which may be very old since they drifted to high MAF. Moreover, the **G**_**LA**_ matrix may focus on too few generations [8]. Hence, in this work, we developed a relationship matrix, **G**_**ROH**_, based on runs of homozygosity (ROH), which considers a range of relationship ages that is between that considered by **G**_**IBS**_ and **G**_**LA**_.

For **G**_**ROH**_, IBD probabilities are calculated using a multi-locus measure of LD called ROH or haplotype homozygosity [10]. ROH is defined as the probability that all consecutive markers on a pair of homologous chromosome segments, in the same or different individual(s), have identical alleles, which indicates IBD [11]. The probability of IBD can be calculated from the distribution of the length of homozygous chromosome segments that surround an IBD locus, since the mean of this length is approximately (log *N*_*e*_ – 1)/2 *N*_*e*_, where *N*_*e*_ is the effective population size [12]. Hayes et al. [11] found that ROH over long distances reflects recent *N*_*e*_, whereas ROH over small distances reflects the *N*_*e*_ in the more distant past. ROH can be used to measure multi-locus LD between a marker and a QTL. Compared to two-locus measures of LD such as *r*^2^, a major advantage of ROH is that it is generally a less variable indicator of IBD than *r*^2^, since the latter is known to be very variable [13]. The variability of LD measured by ROH decreases as the marker density increases, whereas variability of LD based on *r*^2^ is unaffected by the number of markers [11].

In this paper, we propose a novel method to predict GEBV based on ROH, hereafter referred to as **G**_**ROH**_. Using simulated datasets with various thresholds of MAF for markers and QTL, we compared the accuracy of GEBV prediction using **G**_**ROH,**_**G**_**IBS**_ based on (population-wide) LD, and **G**_**LA**_ based on linkage analysis. In addition, we evaluated the accuracy of prediction of these methods using real data of deregressed EBV of 1086 Italian Brown Swiss bulls.

## Methods

### Simulation

A forward simulator (http://ihaiwtheoserv.umb.no/tools/xform/xform.tar.gz) was used to simulate populations according to Wright’s ideal population model, i.e. with random mating, uniform mutation rate and base pair position, drift/mutation balance, manageable effective size, SNP mutations that are accumulated through generations of spontaneous mutations and recombinations under random mating. The ideal populations had an effective size of 500, a 1:1 sex ratio and a mutation rate of 10^−8^ per base pair per meiosis. To maintain a reasonable computation time, only one chromosome of length 1 Morgan was simulated.

After 10 000 generations of random mating, the genotypes of the newly produced individuals, referred to as generation 0, were recorded. Genotypes for two kinds of pedigrees were created with generation 0, and were used to produce two simulated datasets i.e. Data I and Data II. Data I was based on a sampled pedigree that was based on 25 sires randomly sampled from the previous generation that were randomly mated to 250 dams randomly sampled from the same generation. Each dam had two offspring. This procedure was repeated for eight generations. Genotypes of the last five generations were recorded to form the simulated dataset. The simulation was performed 10 times to obtain 10 replicates of Data I.

For Data II, the genotypes after 10 000 generations of random mating were gene-dropped through a real pedigree of the Italian Brown Swiss population. The population consisted of 11 599 animals, including 3626 founders and their offspring. There were 27 generations in the pedigree. Genotypes simulated for generation 0 were diffused into this pedigree through its founders. The simulated genotypes of the individuals that were genotyped in the real data were recorded to obtain Data II. This simulation was also performed 10 times to obtain 10 replicates.

One thousand SNPs per chromosome and 30 QTL were sampled disjointedly (i.e. QTL loci could not be sampled as marker loci) from the genotypes created above. Five sampling strategies were used according to the SNP allele frequencies to obtain the following five populations in each dataset (Data I and Data II): Population 1 consisted of randomly sampled markers and QTL (MAF_SNP_ > 0, MAF_QTL_ > 0); Population 2 consisted of markers all sampled with a minimum MAF of 0.1 and QTL with a maximum MAF of 0.1 (MAF_SNP_ > 0.1, MAF_QTL_ < 0.1); Population 3 consisted of markers with a minimum MAF of 0.1 and QTL sampled at random (MAF_SNP_ > 0.1, MAF_QTL_ > 0); Population 4 consisted of markers sampled at random and QTL sampled with a maximum MAF of 0.1 (MAF_SNP_ > 0, MAF_QTL_ < 0.1); Population 5 consisted of markers with a minimum MAF of 0.15 and QTL sampled with a maximum MAF of 0.05 (MAF_SNP_ > 0.15, MAF_QTL_ < 0.05). These five populations reflect the varying degrees to which SNPs can be selected for inclusion on the SNP chip based on high MAF and the variable low frequency of QTL due to selection.

The simulated QTL effects were additive and followed a Laplacian distribution with mean 0 and shape parameter 1. The phenotypes were finally simulated by adding random environmental effects that were independently, identically and normally distributed, in order to achieve a heritability of 0.10.

### Real data

The real data on 1086 Italian Brown Swiss bulls consisted of genotyping (i.e. 35 706 SNPs) and phenotyping data i.e. de-regressed proofs (DP) for three traits: milk yield (kg), milk fat yield (kg) and milk protein yield (kg). A detailed description of these data is reported in Luan et al. [8].

### Cross-validation data

To obtain the cross-validation datasets, the phenotypes of a defined number of individuals were masked. For the simulated data, in Data I we randomly selected 500 of 4500 individuals at a time for each replicate, without replacement, to produce nine non-overlapping cross-validation datasets, i.e., every phenotype was masked once. Therefore, a total of 90 cross-validation datasets were produced for 10 replicates. Similarly, in Data II we randomly selected 181 of 1086 individuals at a time to produce six non-overlapping cross-validation datasets for each replicate, resulting in 60 datasets. The GEBV of the masked individuals were predicted by the genomic prediction methods described in the next section. The correlation coefficient between the GEBV and true breeding values (TBV) was calculated and used as a measure of the GEBV prediction accuracy, and the deviation of the coefficient of regression of TBV on GEBV from 1 was used as a measure of bias. The mean and standard error of the prediction accuracies and biases in the 90 and 60 datasets for Data I and Data II, respectively, were calculated for each population and each prediction method.

For the analysis with real data on 1086 Italian Brown Swiss bulls, two strategies were used to produce cross-validation datasets. The first strategy was the same as that applied to Data II, except that the GEBV were correlated to the masked DP, to obtain a measure of the accuracy of the GEBV (this measure does not have a maximum of 1 since the reliability of the DP is less than 100%). To obtain standard errors, the division into sets and GEBV predictions were replicated 10 times. The second strategy consisted of selecting the youngest bulls as the validation dataset. In practice, to obtain a validation dataset of reasonable size, we selected bulls born in the three most recent years in the pedigree (2003, 2004 and 2005). One hundred and sixteen young bulls were selected, and their GEBV were predicted using data on 970 older animals.

### ROH-based relationships

A run of homozygosity is defined as two haplotypes carrying IBS marker alleles from some position i through to some position j. Let ROH(i,j) denote the probability of this occurring (without making any assumptions about marker identity at the border positions (i-1) and (j + 1)). The method to calculate ROH(i,j) was described in detail by Macleod et al. [10]. Briefly, it calculates the probability that no mutation at the marker positions has occurred since the two homologous chromosome segments coalesced into a common ancestor, integrated over all possible coalescence times. The calculations also account for the fact that the segment between markers i and j may consist of a combination of several shorter IBD segments that each coalesced into different common ancestors. The calculations require knowledge on the genetic distances between the markers, their mutation rates, and the effective population size (*N*_*e*_), which was assumed to be 100. An approximate estimate for the mutation rate at the markers, m_m_, is obtained by equating the average homozygosity of all markers to its expected value 1/(1 + 4*N*_*e*_m_m_). Here, we will also consider run of homozygosity probability ROH(i,k,j), where a putative focal position k which is in the middle between two consecutive markers forming a marker bracket, is also assumed to be IBS (with i < k < j). Since the focal position k is in the middle between two markers, there is no actual marker data at this position. Thus, ROH(i,j) is the sum of the probability ROH(i,k,j) and the probability that all positions except position k carry IBS alleles. Let ROH(−i,−j) denote the probability that all marker alleles between positions i and j are IBS between two haplotypes, but the haplotypes are not IBS at positions (i−1) and (j + 1) (for ROH, we usually observe that all markers between positions i and j are IBS but that IBS does not extend beyond the boundaries i and j). Bounded ROH probabilities can be calculated from unbounded ROH(i,j) probabilities as [14]:

$$ \mathrm{R}\mathrm{O}\mathrm{H}\left(-\mathrm{i},-\mathrm{j}\right)=\mathrm{R}\mathrm{O}\mathrm{H}\left(\mathrm{i},\mathrm{j}\right)-\mathrm{R}\mathrm{O}\mathrm{H}\left(\mathrm{i}-1,\mathrm{j}\right)-\mathrm{R}\mathrm{O}\mathrm{H}\left(\mathrm{i},\mathrm{j}+1\right)+\mathrm{R}\mathrm{O}\mathrm{H}\left(\mathrm{i}-1,\mathrm{j}+1\right). $$

Inclusion of an extra position k among the IBS markers is straightforward:

$$ \mathrm{R}\mathrm{O}\mathrm{H}\left(-\mathrm{i},\mathrm{k},-\mathrm{j}\right)=\mathrm{R}\mathrm{O}\mathrm{H}\left(\mathrm{i},\mathrm{k},\mathrm{j}\right)-\mathrm{R}\mathrm{O}\mathrm{H}\left(\mathrm{i}-1,\mathrm{k},\mathrm{j}\right)-\mathrm{R}\mathrm{O}\mathrm{H}\left(\mathrm{i},\mathrm{k},\mathrm{j}+1\right)+\mathrm{R}\mathrm{O}\mathrm{H}\left(\mathrm{i}-1,\mathrm{k},\mathrm{j}+1\right). $$

Now, given that we know that all actual markers are IBS between positions i and j and not IBS at positions (i−1) and (j + 1), the IBD probability at position k is defined as the probability that there has been no mutation at this position since its coalescence:

$$ \mathrm{PIBD}\left(\mathrm{k}\left|-\mathrm{i},-\mathrm{j}\right.\right)=\mathrm{R}\mathrm{O}\mathrm{H}\left(-\mathrm{i},\mathrm{k},-\mathrm{j}\right)/\mathrm{R}\mathrm{O}\mathrm{H}\left(-\mathrm{i},-\mathrm{j}\right). $$

Here, we need to make an (arbitrary) assumption about the mutation rate at position k (m_k_), which is chosen such that the a priori IBD probability at position k is close to 0.5, i.e. 1/(1 + 4*N*_*e*_m_k_) ≈ 0.5, in order to give the marker data ample opportunity to change the a priori probability either towards 0 (few or no IBS markers in the vicinity of k) or towards 1 (k is in the middle of a long stretch of IBS markers).

IBD probability PIBD(k|−i,−j) is calculated and averaged over all marker brackets in the genome, with the focal position k in the middle of each bracket. The averaged PIBD(k|−i,−j) of all combinations of genotyped animals are stored in a ROH-based relationship matrix, called **G**_**ROH**_. **G**_**ROH**_ is not always positive definite, because its elements are calculated on a one-by-one basis. Therefore, the eigenvalues of **G**_**ROH**_ are checked, negative eigenvalues are set to 0, and the matrix is reconstructed using only the positive eigenvalues. Finally, a small value (0.0001) is added to the diagonals to make **G**_**ROH**_ positive definite.

The calculation of PIBD(k|−i,−j) and **G**_**ROH**_ is implemented in the LDMIP software (http://ihaiwtheoserv.umb.no/tools/ldmip) [15]. Program LDMIP can also use the PIBD(k|−i,−j) probabilities for imputation of missing marker data, i.e. it finds the N_hap_ haplotypes that resemble the haplotype with a missing marker based on the highest PIBD probability at every position k. Next, it uses the Viterbi algorithm [16] to find, for the current haplotype, a path through these N_hap_ haplotypes without mismatches between the current and the proposed haplotype and with the fewest number of switches between the N_hap_ haplotypes. I.e., the algorithm finds a mosaic of the N_hap_ haplotypes that most closely resembles the current haplotype, and uses this mosaic to impute the missing markers. Because marker phase is often unknown (i.e. a heterozygous genotype is not known to be ‘1 2’ or ‘2 1’), the Viterbi algorithm is actually applied to resolve both haplotypes of an individual simultaneously, resulting in a mosaic (as explained above) for each of the two haplotypes and resolving the phase of heterozygous genotypes (‘1 2’ or ‘2 1’). For this, the Viterbi algorithm considers N_hap_^2^ combinations of the N_hap_ haplotypes that were selected based on PIBD. Based on some preliminary testing, we found N_hap_ = 40 as a suitable tradeoff between accuracy and computing time. The LDMIP algorithm also yields probabilities of paternal and maternal inheritance at the marker alleles for all animals in the pedigree [15], which can be used to set up a linkage analysis based on the genomic relationship matrix **G**_**LA**_ by setting up such a relationship matrix at all marker positions and averaging across positions [8,9].

### GEBV prediction based on IBS, LA and ROH relationships

The model used to predict GEBV with IBS, LA and ROH information can be expressed as:

$$ \mathbf{y}=\mathbf{1}\mu \kern0.5em +\kern0.5em \mathbf{Z} \mathbf{a}\kern0.5em +\kern0.5em \mathbf{e}, $$

where **y** is a vector of phenotypes (DP) for a trait; *μ* is the overall mean; **Z** is a design matrix linking the animals to the phenotypes; **a** is a vector of additive genetic effects of the animals and **e** is the vector of random residuals. It is assumed that **a**_*(.)*_ ~ *N* (**0**, **G**_**(.)**_*σ*_*(.)*_^*2*^) where (.) refers to ROH, LA or IBS and *σ*_*(.)*_^*2*^ is the additive genetic variance associated with **G**_**(.)**_.

For GEBV prediction based on IBS relationships, **G**_**(.)**_ is **G**_**IBS**_. Two ways to set up the **G**_**IBS**_ matrix were used here. The first was to construct the **G**_**IBS**_ matrix with standardized marker genotypes (**G**_**IBS-STD**_) as **G**_**IBS-STD**_ = **XX’/***N*_*m*_, where *N*_*m*_ is the number of markers and **X** is a matrix of the standardized marker genotypes, ${X}_{ij}=\left({\mathit{\mathsf{g}}}_{ij}-2{p}_j\right)/\sqrt{2{p}_j\left(1-{p}_j\right)}$, where *g*_*ij*_ is the genotype of animal *i* for SNP *j*, with *g*_*ij*_ = 0, 1 or 2 for genotypes “0 0”, “1 0” or “1 1”, respectively, and *p*_*j*_ is the frequency of allele 1 of SNP *j*. Standardization is such that the mean and the variance of *X*_*ij*_ are 0 and 1, respectively [6]. The second method used to construct the **G**_**IBS**_ matrix was as in VanRaden [5], where markers are not standardized and the IBS matrix is calculated as **G**_**IBS** − **NSTD**_ = **YY** ’/∑(2*p*_*j*_(1 − *p*_*j*_), where **Y** is a matrix of non-standardized marker genotypes, i.e. ${Y}_{ij}={\mathit{\mathsf{g}}}_{ij}-2{p}_j$.

For GEBV prediction based on LA relationships, **G**_**(.)**_ is **G**_**LA**_, the LA-based genomic IBD relationship matrix. For a detailed description about models for GEBV prediction based on LA relationships, see Luan et al. [8]. For GEBV prediction based on ROH relationships, **G**_**(.)**_ is **G**_**ROH**_, the ROH-based genomic IBD relationship matrix. To implement the models, **G**_**IBS-STD**_, **G**_**IBS-NSTD**_, **G**_**LA**_ and **G**_**ROH**_ were inverted and were then used in ASReml [17] to predict GEBV of both phenotyped and non-phenotyped individuals.

## Results

### Accuracy and bias of GEBV prediction using simulated data

We evaluated the accuracy and bias of the GEBV obtained by **G**_**ROH**_ in 90 simulated datasets of Data I and in 60 datasets of Data II and compared them with GEBV based on **G**_**IBS-STD**_, **G**_**IBS-NSTD**_ and **G**_**LA**_ relationship matrices. Means and standard errors of the accuracies and biases of the GEBV are in Tables 1 and 2 for Data I and II, respectively.

**Table 1:** **Means and standard errors of accuracies and biases of GEBV obtained from four methods for populations with different thresholds of MAF for markers and QTL in Data I**

**MAF**		**Method**			
**SNP**	**QTL**	**G**_**ROH**_	**G**_**LA**_	**G**_**IBS-STD**_	**G**_**IBS-NSTD**_
**Accuracy**					
> 0	> 0	0.750 _± 0.003_	0.676 _± 0.004_	0.725 _± 0.005_	0.720 _± 0.005_
> 0.10	< 0.10	0.704 _± 0.004_	0.623 _± 0.006_	0.669 _± 0.005_	0.665 _± 0.005_
> 0.10	> 0	0.760 _± 0.004_	0.669 _± 0.005_	0.751 _± 0.005_	0.751 _± 0.005_
> 0	< 0.10	0.721 _± 0.003_	0.649 _± 0.004_	0.671 _± 0.005_	0.661 _± 0.005_
> 0.15	< 0.05	0.699 _± 0.005_	0.643 _± 0.007_	0.653 _± 0.006_	0.652 _± 0.006_
**Bias** ^**1**^					
> 0	> 0	1.017 _± 0.007_	1.005 _± 0.009_	1.009 _± 0.008_	1.006 _± 0.007_
> 0.10	< 0.10	1.002 _± 0.010_	0.984 _± 0.011_	1.014 _± 0.011_	1.013 _± 0.012_
> 0.10	> 0	1.020 _± 0.010_	1.030 _± 0.011_	1.023 _± 0.010_	1.023 _± 0.010_
> 0	< 0.10	1.029 _± 0.010_	1.025 _± 0.011_	1.043 _± 0.012_	1.039 _± 0.013_
> 0.15	< 0.05	1.026 _± 0.013_	1.056 _± 0.017_	1.021 _± 0.014_	1.021 _± 0.014_

^1^Bias is calculated as the regression of TBV on GEBV; bias is equal to 1 if the GEBV prediction is unbiased.

**Table 2:** **Mean and standard error of the accuracies and biases of GEBV prediction for populations with different thresholds of MAF for markers and QTL in Data II**

**MAF**		**Method**			
**SNP**	**QTL**	**G**_**ROH**_	**G**_**LA**_	**G**_**IBS-STD**_	**G**_**IBS-NSTD**_
**Accuracy**					
> 0	> 0	0.600 _± 0.009_	0.570 _± 0.010_	0.582 _± 0.011_	0.579 _± 0.011_
> 0.10	< 0.10	0.466 _± 0.013_	0.457 _± 0.012_	0.425 _± 0.012_	0.424 _± 0.012_
> 0.10	> 0	0.603 _± 0.008_	0.571 _± 0.009_	0.597 _± 0.009_	0.594 _± 0.009_
> 0	< 0.10	0.533 _± 0.016_	0.528 _± 0.016_	0.496 _± 0.017_	0.490 _± 0.017_
> 0.15	< 0.05	0.406 _± 0.015_	0.395 _± 0.016_	0.369 _± 0.015_	0.368 _± 0.015_
**Bias** ^**1**^					
> 0	> 0	1.101 _± 0.041_	1.055 _± 0.037_	1.114 _± 0.045_	1.137 _± 0.051_
> 0.10	< 0.10	1.327 _± 0.149_	1.357 _± 0.131_	1.364 _± 0.153_	1.393 _± 0.170_
> 0.10	> 0	0.961 _± 0.026_	0.983 _± 0.033_	0.955 _± 0.030_	0.948 _± 0.030_
> 0	< 0.10	1.140 _± 0.052_	1.110 _± 0.049_	1.157 _± 0.056_	1.225 _± 0.064_
> 0.15	< 0.05	1.479 _± 0.215_	1.795 _± 0.356_	1.776 _± 0.286_	1.728 _± 0.271_

^1^Bias is calculated as the regression of TBV on GEBV.

For all datasets, accuracy of the GEBV was higher with **G**_**ROH**_ than with **G**_**LA**_, **G**_**IBS-STD**_ and **G**_**IBS-NSTD**_, although the differences were not always statistically significant. Accuracies of GEBV based on **G**_**IBS-STD**_ and **G**_**IBS-NSTD**_ were similar. Accuracies of GEBV were higher and biases lower with Data I (Table 1) than with Data II (Table 2), which is expected since the size of the reference population was larger in Data I (4000 individuals) than in Data II (905 individuals). It is also notable that the presence of rare QTL alleles reduced the accuracy much more with real pedigree structures (Data II) than with random pedigrees (Data I). For the simulated dataset with a random pedigree (Data I), accuracies were higher with **G**_**LA**_ than with **G**_**IBS**_ for all QTL allele frequency scenarios (Table 1). For the simulated dataset with real pedigree (Data II), accuracies were also higher with **G**_**LA**_ than with **G**_**IBS**_ when a maximum MAF was applied to the QTL but were lower when QTL were randomly sampled (Table 2).

Results from simulations (Tables 1 and 2) demonstrated that the accuracy of GEBV was affected by the MAF of markers and QTL. The highest accuracy was obtained in the population with a minimum MAF of 0.1 for markers and no MAF threshold applied to QTL. Application of a maximum MAF threshold to QTL appeared to reduce the accuracy of GEBV. For example, for GEBV using **G**_**ROH**_, the accuracies decreased by ~38% and ~11% when QTL had a MAF below 0.1 in Data I and Data II, respectively, compared to when both QTL and SNPs were randomly sampled. When a minimum MAF was applied to markers and a maximum MAF to QTL, accuracies of GEBV were reduced.

### Correlation between GEBV and DP and bias of GEBV using real data

To investigate the performance of the **G**_**ROH**_-based method in practice, we applied **G**_**ROH**_ to real DP datasets of 1086 Italian Brown Swiss bulls for fat yield, milk yield and protein yield. Table 3 presents the correlations between GEBV and DP and biases of GEBV with **G**_**ROH**_, **G**_**IBS-STD,**_**G**_**IBS-NSTD**_ and **G**_**LA**_, since the TBV is unknown in the real dataset. The bias was calculated as the regression of DP on GEBV. However, it should be noted here that any under- or over-scaling of the DP by the deregression process will appear as regression coefficients that deviate from 1, i.e. as bias [8]. Table 3 shows that the correlations obtained with **G**_**ROH**_ were higher than those with **G**_**IBS**_ and very similar to those with **G**_**LA**_ but with a substantially lower regression coefficient. In agreement with results from the simulated data, the **G**_**IBS-STD**_**-** and **G**_**IBS-NSTD**_-based methods resulted in similar correlations and biases. The correlation obtained for protein yield was higher than that for fat and milk yields. The standard error of the correlations obtained with the **G**_**ROH**_-based method was smaller than that of the other methods, which indicates that the results were less variable. This is in line with the expectation of Hayes et al. [11] that multi-locus measures of LD are less variable.

**Table 3:** **Mean and standard error of the correlation between GEBV and DP, and biases of the GEBV evaluated with cross-validation in real data**

**Trait**		**G**_**ROH**_	**G**_**LA**_	**G**_**IBS-STD**_	**G**_**IBS-NSTD**_
**Correlation**					
Fat yield		0.768 _± 0.007_	0.768 _± 0.010_	0.751 _± 0.010_	0.752 _± 0.009_
Milk yield		0.763 _± 0.007_	0.762 _± 0.010_	0.748 _± 0.009_	0.748 _± 0.009_
Protein yield		0.784 _± 0.007_	0.784 _± 0.010_	0.768 _± 0.009_	0.767 _± 0.009_
**Bias** ^**1**^					
Fat yield		1.002 _± 0.031_	1.132 _± 0.026_	1.022 _± 0.026_	1.015 _± 0.027_
Milk yield		1.004 _± 0.024_	1.124 _± 0.020_	1.026 _± 0.021_	1.018 _± 0.021_
Protein yield		1.009 _± 0.023_	1.130 _± 0.020_	1.036 _± 0.020_	1.027 _± 0.020_

^1^Bias is calculated as the regression of DP on GEBV.

**Figure 1:**
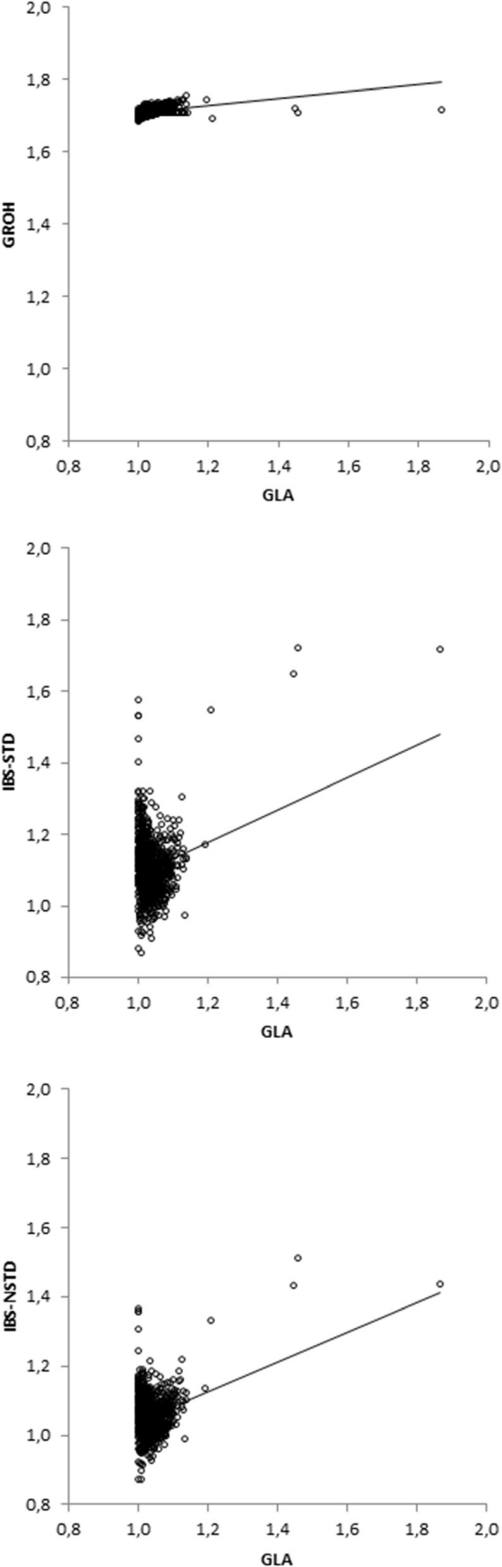
**Scatter plot of diagonal entries of G**_**ROH**_ **matrix and G**_**IBS**_ **matrices versus G**_**LA**_ **matrix.**

**Figure 2:**
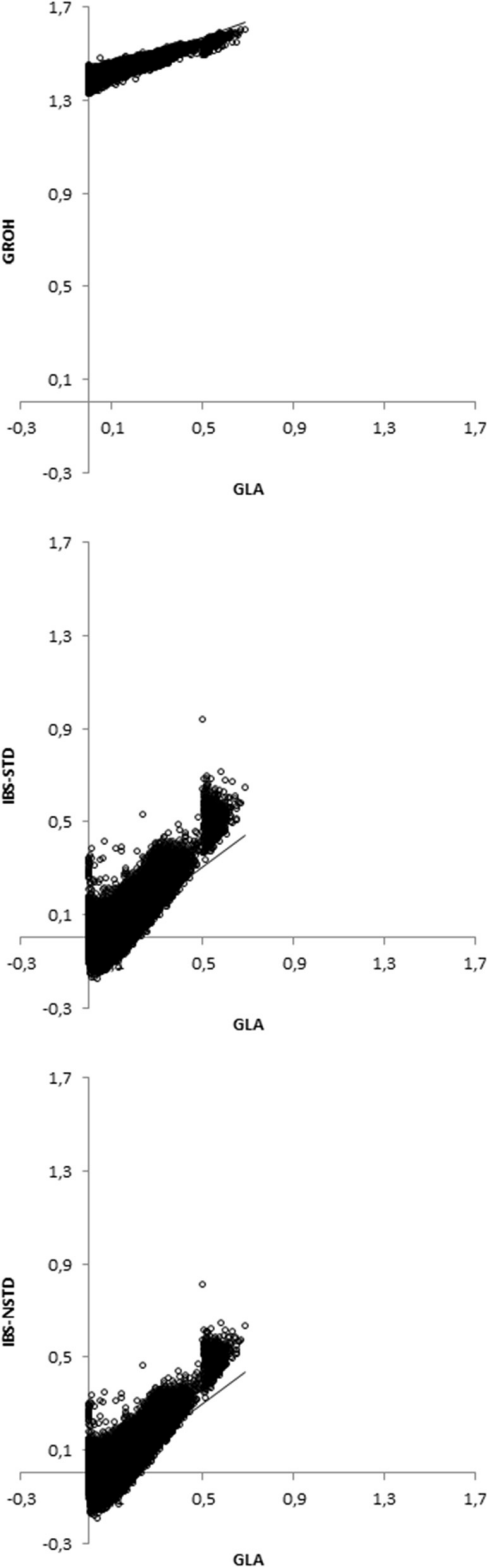
**Scatter plots of off-diagonal entries of G**_**ROH**_ **matrix and G**_**IBS**_ **matrices versus G**_**LA**_ **matrix.**

Correlations between GEBV and DP and biases of GEBV obtained when **G**_**ROH**_, **G**_**LA**_ and **G**_**IBS**_ were used to predict the group of 116 young bulls are in Table 4. In contrast to the analyses with randomly selected cross-validation bulls, GEBV prediction for the group of young bulls was performed only once and thus no standard errors were available. The correlation between GEBV and DP was higher with **G**_**LA**_ than with **G**_**ROH**_ and **G**_**IBS**_ (Table 4). Also, the **G**_**IBS-NSTD**_-based method resulted in slightly higher correlations than the **G**_**IBS-STD**_-based method. Figures 1 and 2 show the scatter-plots of 1086 diagonal and 589 156 off-diagonal entries of **G**_**ROH**_, **G**_**IBS-STD**_ and **G**_**IBS-NSTD**_ versus the **G**_**LA**_ matrix for the real data. Regression lines of the entries of **G**_**ROH**_, **G**_**IBS-STD**_ and **G**_**IBS-NSTD**_ matrix on those of the **G**_**LA**_ matrix are also shown in these figures.

**Table 4:** **The correlation between GEBV and DP, and biases of GEBV evaluated for the group of young animals in the real data**

**Trait**	**G**_**ROH**_	**G**_**LA**_	**G**_**IBS-STD**_	**G**_**IBS-NSTD**_
**Correlation**				
Fat yield	0.447	0.500	0.400	0.415
Milk yield	0.410	0.415	0.376	0.389
Protein yield	0.385	0.410	0.363	0.368
**Bias** ^**1**^				
Fat yield	0.946	1.277	0.835	0.855
Milk yield	0.829	1.013	0.778	0.789
Protein yield	0.763	1.007	0.736	0.733

^1^Bias is calculated as the regression of DP on GEBV.

## Discussion

In this study, we proposed **G**_**ROH**_-based genomic prediction, a novel method to compute GEBV based on runs of homozygosity. Runs of homozygosity yield a multi-locus measure of LD, from which a measure of IBD is derived, which we expected to be less variable than the IBD derived by single-locus measures of LD and thus to result in an increased accuracy of GEBV. Using simulated and real data, the accuracy and bias of GEBV based on **G**_**ROH**_ were compared to those based on **G**_**LA**_ and **G**_**IBS**_. Results from simulation analyses showed that, in general, **G**_**ROH**_ resulted in more accurate GEBV, up to 4% points higher. With the real data, the accuracy of GEBV was higher with **G**_**ROH**_ than with **G**_**IBS**_ but only slightly higher than with **G**_**LA**_. Predictions using **G**_**ROH**_ and, especially, **G**_**LA**_ were less affected by the difference in allele frequencies between QTL and markers, probably because they do not rely on pair-wise LD between QTL and markers. A possible explanation of the difference in results between real and simulated data may lie in the difference in the population structures in the datasets. In the real dataset, we found that recent family relationships are strong in the population of bulls used [8]. Thus, in the real data, older and more distant relationships may contribute little to the accuracy of GEBV. This favors **G**_**LA**_, which relies on recent family relationships to predict GEBV, whereas **G**_**IBS**_ relies on more distant relationships and hence yielded lower accuracies than **G**_**LA**_ for the real data. Matrix **G**_**ROH**_ captures relationships that span a range of ages of relationships that is intermediate between those captured by **G**_**LA**_ and **G**_**IBS**_. Matrix **G**_**ROH**_ also takes recent family relationships much more in account and resulted in accuracies of GEBV that were as high as with **G**_**LA**_ for the real data. In simulated data based on a sampled pedigree (Data I), the older and more distant relationships contributed more to the accuracy of GEBV, which favors **G**_**IBS**_ and thus the accuracy obtained with **G**_**IBS**_ was higher than with **G**_**LA**_ for Data I.

For the simulated data based on the real pedigree from the population with strong recent family relationships (Data II), the results in Table 2 show that the performance of **G**_**LA**_ and **G**_**IBS**_ depended on the simulation scenario. If QTL were randomly sampled, the older and more distant relationships were important because this scenario allows old QTL mutations to still contribute to the genetic variance. Matrix **G**_**IBS**_ can capture such relationships and hence achieved higher accuracy than **G**_**LA**_ for that scenario. If QTL were sampled with a maximum threshold of MAF, then mainly recent QTL mutations contribute to the genetic variance. This implies that recent family relationships are important for the accuracy of GEBV and that more distant relationships contribute little, as indicated by the fact that **G**_**LA**_ achieved higher accuracy than **G**_**IBS**_ for this scenario (Table 2).

In the simulation results, **G**_**ROH**_ achieved higher accuracies than **G**_**LA**_ and **G**_**IBS**_ in all cases. In cases where older and more distant relationships are important (Table 1 and MAF_QTL_ > 0 in Table 2), **G**_**IBS**_ can capture such relationships and, therefore, achieved higher accuracies than **G**_**LA**_. However, the capacity of **G**_**IBS**_ to capture information on old relationships depends on the use of uncertain relationships between ancestors, which may undermine the performance of **G**_**IBS**_. In contrast, **G**_**ROH**_ does not look back as many generations as **G**_**IBS**_ and thus yields higher accuracies than **G**_**IBS**_. In cases where recent family relationships are important (QTL sampled with a MAF threshold in Table 2), **G**_**LA**_ achieved higher accuracies than **G**_**IBS**_. The capacity of **G**_**ROH**_ to capture non-recorded relationships before the base generation of the known pedigree means that **G**_**ROH**_ can use such information and thus achieve higher accuracies than **G**_**LA**_. The latter would be especially useful for across-breed prediction, as in the case when the training population contains several breeds. Thus, it appears that our novel method **G**_**ROH**_ can benefit from the favorable properties of both **G**_**LA**_ and **G**_**IBS**_.

The relationship matrices used in this study differ in the chromosomal distance they consider. Matrix **G**_**LA**_ can consider a distance up to one complete chromosome, while **G**_**IBS**_ relies on pair-wise LD between markers and QTL, which stretches only over small chromosomal distances. Matrix **G**_**ROH**_ uses multi-locus LD and thus can account for larger chromosomal distances to capture LD than **G**_**IBS**_. Matrix **G**_**ROH**_ seems to strike a balance between short and long range LD and resulted in the highest prediction accuracies for a range of situations, except for prediction of the youngest animals in the data set, which is however a typical scenario for genomic selection in practice.

Similar to the way both marker and pedigree information are used in matrix **G**_**LA**_, Goddard et al. [4] proposed a method to obtain an unbiased estimate of relationships for genomic prediction by regressing the IBS matrix onto the pedigree-based relationship matrix. Their method uses the relationship matrix $\widehat{\mathbf{G}}=\mathbf{A}+b\left({\mathbf{G}}_{\mathbf{IBS}\mathbf{\hbox{-}}\mathbf{S}\mathbf{T}\mathbf{D}}-\mathbf{A}\right)$ for genomic prediction, where **A** is the relationship matrix based on pedigree, and the regression coefficient *b* reflects the proportion of genetic variance explained by the markers. This method attempts to take the whole range of population structures into consideration: matrix **A** accounts for recent relationships and matrix **G**_**IBS-STD**_ for distant relationships. Therefore, when recent family relationships are more important, use of matrix $\widehat{\mathbf{G}}$ may yield higher accuracies than use of **G**_**IBS**_. The regression coefficient *b* depends on marker density and putative differences in the properties of markers vs. QTL. If QTL and markers do not systematically differ, for example markers and QTL are randomly sampled, as explored in this study, *b* can be predicted from the marker data. If QTL and markers differ systematically (e.g. MAF_SNP_ > 0.15 and MAF_QTL_ < 0.05 in this study), the regression coefficient *b* should be estimated from the data, which can be achieved by fitting the **A** and **G**_**IBS-STD**_ matrices jointly in a variance component estimation model.

Accuracies were lower for prediction of young animals than for random cross-validation datasets. This may be due to the fact that the Mendelian sampling component of their TBV, i.e. about half of the total variance of the TBV, is uncorrelated to any of the training records. The results for the group of young animals in the real data show that the highest accuracy was obtained with **G**_**LA**_, followed by **G**_**ROH**_ and **G**_**IBS**_. A possible explanation may be that the three methods capture different relationship ages. Matrix **G**_**LA**_ only focuses on the known pedigree, for which the relationships of young animals can be well-defined. With **G**_**IBS**_, uncertain relationships between ancestors prior to the known pedigree may deteriorate the ability to capture relationships between young animals. Matrix **G**_**ROH**_ captures information on relationships for ages that are intermediate between those of **G**_**LA**_ and **G**_**ROH**_, and thus achieves an intermediate accuracy.

The performance of the methods also depends on the effective size of the population (*N*_*e*_). If *N*_*e*_ is small, common ancestors tend to be in the recent past and recent family relationships tend to dominate the population structure. It is expected that **G**_**LA**_ performs better than **G**_**ROH**_. If *N*_*e*_ is large, distant family relationships occur frequently and the performance of **G**_**LA**_ deteriorates. Thus, it is expected that **G**_**LA**_ will perform worse than **G**_**ROH**_ in a population with a large *N*_*e*_ and when the training population consists of a mixture of different breeds. In the analysis with real data, **G**_**LA**_ was found to give higher accuracies than **G**_**ROH**_, while in the simulation study with *N*_*e*_ = 500, **G**_**ROH**_ performed better. This suggests that the Italian Brown Swiss bull population has a smaller *N*_*e*_ than 500, which is also suggested by its small number of sires (21). Goddard et al. [4] pointed out that variation in relationships between animals in a population increases with *N*_*e*_. Thus, **G**_**ROH**_ is expected to result in higher accuracies of prediction than **G**_**LA**_ when variation in relationships is small, such as between breeds.

In the simulation study, we used two methods to calculate the matrix **G**_**IBS**_, which differed in whether markers were standardized or not prior to its calculation. It is known that standardizing markers increases the weight placed on low MAF markers [18]. The effects of markers with low MAF are estimated with much lower accuracy. This suggests that the standardization of markers may result in different accuracies. However, our results show that the two methods of computing **G**_**IBS**_ resulted in similar accuracies. This agrees with the expectation of Sonesson et al. [18].

The simulated and real data results were quite different even when the real pedigree was used in the simulations. The changes in allele frequencies between markers and QTL introduced in Table 2 did not result in simulated results being closer to the real data results of Table 3. Possibly, in the real data, the QTL do not have a MAF as low as that simulated in Table 2 because they have been recently selected and the population has high rates of inbreeding, which causes low allele frequencies to drift towards intermediate values. It seems that LA information was much more important for the analysis of real data than that of simulated data. A possible explanation is the much higher reliability of the deregressed proofs compared to that of the simulated trait (h^2^ = 0.1), which also resulted in the higher cross-validation accuracies in the real data. This high reliability of the de-regressed proofs resulted in accurate estimation of chromosomal segments in the linkage analysis, while the low heritability of the simulated trait implies that the long-term, LD-based genetic effects also need to be estimated to achieve high cross-validation accuracy.

## Conclusions

The present study proposes a novel method, **G**_**ROH**_, to predict GEBV based on runs of homozygosity. Through computer simulations, we showed that the accuracy of GEBV was higher with **G**_**ROH**_ than with **G**_**IBS**_ and **G**_**LA**_. In the analyses of real data, accuracies obtained with **G**_**ROH**_ and **G**_**LA**_ were similar and for the youngest animals, they were highest with **G**_**LA**_. The accuracies obtained with the LD matrix calculated with or without standardizing marker genotypes were similar.
